# Artery of Percheron Occlusion in an Elderly Male: A Case Report

**DOI:** 10.14740/jocmr2009w

**Published:** 2014-11-19

**Authors:** Yuh-Ming Chang, Yang-Kai Fan

**Affiliations:** a Department of Neurology, Hsinchu Mackay Memorial Hospital, Hsinchu City, Taiwan; b Department of Radiology, Hsinchu Mackay Memorial Hospital, Hsinchu City, Taiwan

**Keywords:** Consciousness, Mesencephalon, Stroke, Thalamus, Ophthalmoplegia

## Abstract

Acute bilateral paramedian thalamic and mesencephalic infarcts are uncommon. Occlusion of the artery of Percheron (AOP) is presumed to cause this specific stroke syndrome. However, occlusion of the AOP is rare and early diagnosis is challenging. Here we described a 70-year-old male patient who presented with acute disturbance of consciousness due to acute bilateral paramedian thalamo-mesencephalic infarction secondary to AOP occlusion. Anticoagulant therapy was administered, and his consciousness gradually improved.

## Introduction

Acute bilateral paramedian thalamic and mesencephalic infarcts are uncommon [1]. Occlusion of the artery of Percheron (AOP) is presumed to cause this specific stroke syndrome [1]. AOP is a rare arterial variant that arises from one of the P1 segments of the posterior cerebral artery and provides bilateral arterial blood supply to the paramedian thalami and rostral mesencephalon. AOP occlusion results in acute disturbance of consciousness, often associated with vertical gaze palsy and memory impairment [1, 2]. Early diagnosis of AOP occlusion is challenging. Here we described the clinical and imaging study of AOP occlusion in a 70-year-old male with acute disturbance of consciousness. Although our patient was outside the treatment time window for thrombolytic therapy, he gradually regained consciousness after anticoagulant therapy was administered.

## Case Report

A 70-year-old man presented at the emergency department (ED) after being found unresponsive in bed. He was last seen normal approximately 8 h ago. He had a 10-year history of hypertension, type 2 diabetes mellitus, hyperlipidemia and gout. There was no recent history of fever, headache, seizure, or trauma and no known toxic substance exposure. On arrival at the ED, his body temperature was 35.5 °C, blood pressure 131/61 mm Hg, heart rate 49 beats/min and respiratory rate 17 breaths/min. On neurological examination, he was comatose. His neck was supple and his pupils were anisocoric, with a 4 mm right pupil and a 6 mm left pupil. The pupillary light reflex was absent in both eyes. The vertical oculocephalic reflex was absent, and his left eye did not show adduction with turning of the head to the left. His limbs moved in response to painful stimuli. The rest of examinations were unremarkable.

**Figure 1:**
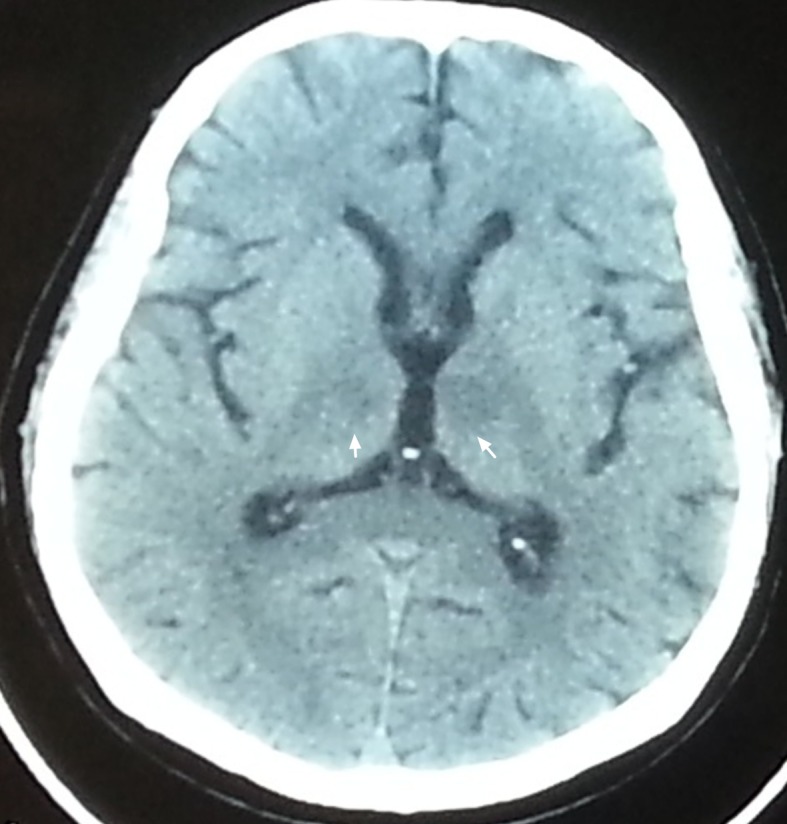
Axial non-contrast brain CT revealed faint hypodense lesion in bilateral paramedian thalamus (arrow).

Laboratory findings including blood glucose, full blood count, electrolytes, liver and renal function tests, thyroid function tests, calcium, arterial blood gas and ammonia were unremarkable. Electrocardiogram (ECG) showed a normal sinus rhythm. Emergent head computed tomography (CT) revealed a faint hypodense lesion in the bilateral paramedian thalamus (Fig. 1). Acute ischemic stroke was suspected and oral aspirin (100 mg/day) was administered.

**Figure 2:**
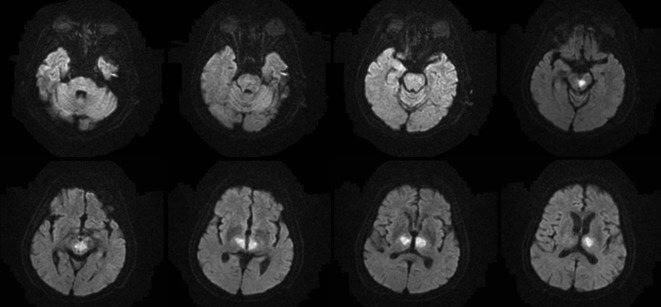
Diffusion-weighted MRI performed on the next day demonstrated hyperintensities in the bilateral thalami and rostral mesencephalon, consistent with restricted diffusion secondary to an acute ischemic stroke in the AOP territory.

**Figure 3:**
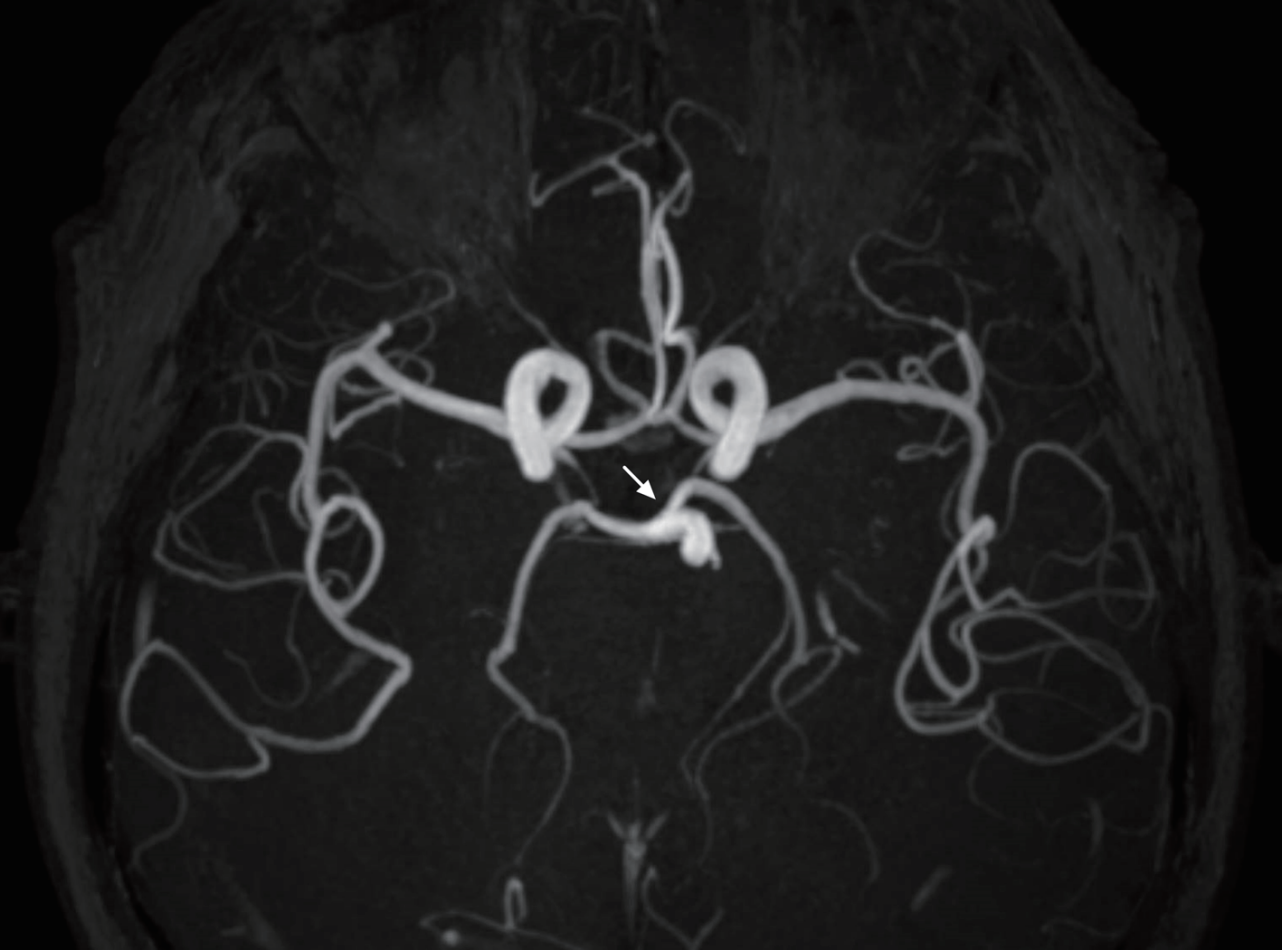
MR angiography demonstrates patent basilar tip and posterior cerebral arteries (arrow).

He was then admitted to the neurointensive care unit. Cerebrospinal fluid analysis was normal. Electroencephalography demonstrated diffuse background slowing without epileptiform activity. Diffusion-weighted magnetic resonance imaging (MRI) performed on the next day demonstrated hyperintensities in the bilateral thalami and rostral mesencephalon, consistent with restricted diffusion secondary to an acute ischemic stroke in the AOP territory (Fig. 2). MR angiography demonstrated patent basilar tip and posterior cerebral arteries (Fig. 3). Anticoagulant therapy was administered. He gradually regained consciousness during the hospitalization days. Upon discharged, his eye sings remained with memory impairment.

## Discussion

Our case illustrates the importance of considering ischemic stroke in the AOP territory in the differential diagnosis of acute disturbance of consciousness in the elderly. The clinical presentation can mimic non-convulsive status epilepticus, subarachnoid hemorrhage, metabolic or toxic encephalopathy, and encephalitis. The low sensitivity of CT makes AOP infarction diagnosis difficult. Diffusion-weighted MRI is the imaging modality of choice [1]. However, AOP is rarely visualized on MR angiography, and lack of visualization does not exclude its presence [1].

AOP prevalence remains unknown. In a recent study, AOP infarction was found in 0.4% patients with a first-ever stroke in their stroke registry [2]. Small-artery disease and cardioembolism were the most frequent stroke mechanisms [2].

These patients must be differentiated from those with “top of the basilar artery” syndrome [1, 3, 4]. However, “top of the basilar artery” syndrome also tends to involve the superior cerebellar artery and posterior cerebral artery territories. The lack of associated lesions in our patient excluded this diagnosis.

Deep cerebral venous thrombosis (DCVT) may also be confused with AOP infarction, because bilateral thalamic infarcts can also result from DCVT [4, 5]. However, DCVT produces simultaneous bilateral thalamic and basal ganglia lesions on MRI that do not respect typical arterial. In addition, MRI patterns usually suggest vasogenic edema rather than arterial infarcts; this is discordant with our patient findings.

Other differential diagnoses considered in our case were hypertensive encephalopathy, Wernicke’s encephalopathy, osmotic demyelination, Japanese encephalitis and Creutzfeldt-Jakob disease. These diagnoses were excluded according to clinical characteristics and imaging features [5].

Successful tissue plasminogen activator therapy for AOP occlusion is reported in the literature [6, 7], but our patient was outside the treatment time window on initial presentation. Li et al suggested that patient with AOP occlusion should receive long-term anticoagulant therapy [8]. Because of the rare occurrence of AOP occlusion, further clinical studies are needed to identify optimal treatment options.

In conclusion, occlusion of the AOP is a rare cause of coma in elderly patients. Diffusion-weighted MRI is the imaging modality of choice for early diagnosis. Early recognition of AOP occlusion may lead to favorable outcomes.
